# Conflit neurovasculaire de l'angle pontocérébelleux

**DOI:** 10.11604/pamj.2014.18.129.3589

**Published:** 2014-06-10

**Authors:** Taoufik Africha, Mohammed Touati

**Affiliations:** 1 Service de Radiologie de l'Hôpital Militaire Moulay Ismail Meknès, Maroc; 2 Service ORL et CCF de l'hôpital militaire Avicenne, Marrakech, Maroc

**Keywords:** Conflit neurovasculaire, angle pontocérébelleux, nerfs crâniens, neurovascular conflict, Cerebellopontine angle, cranial nerves

## Image en medicine

Les conflits neuro-vasculaires (CNV) de l'angle ponto-cérébelleux sont assez rares et peuvent intéresser les Vème, VIIème, VIIIème,IXème paires crâniennes, L'angle ponto-cérébelleux est une région anatomique contenant des éléments vasculo-nerveux, les nerfs crâniens le traversent de façon rectiligne, les vaisseaux y décrivent des trajets très variables et sinueux, cette contiguïté anatomique est responsable de la genèse d'un CNV et l'effet d'usure induit par le battement vasculaire contre la paroi du nerf modifie localement l'histologie et le comportement physiologique de ce dernier. Les CNV se manifestent par des vertiges positionnels, des acouphènes unilatéraux ou une surdité de perception unilatérale progressive. Les potentiels évoqués auditifs (PEA) permettent d’évoquer l'origine nerveuse de la symptomatologie. L'IRM détermine en cas de suspicion de CNV le lieu du conflit, la déformation du nerf et le trajet orthogonal du vaisseau par rapport au trajet du nerf réalisant ainsi une cartographie pré-chirurgicale et l'angio-IRM définit mieux le vaisseau en cause. L'association de signes cliniques, électriques et radiologiques indique la microchirurgie pour décompression par mobilisation du vaisseau responsable et le maintenir à distance du nerf impliqué et le contrôle endoscopique vérifie l'efficacité anatomique du traitement. Le risque majeur par atteinte du nerf est prévenu par monitorage per-opératoire des PEA.

**Figure 1:**
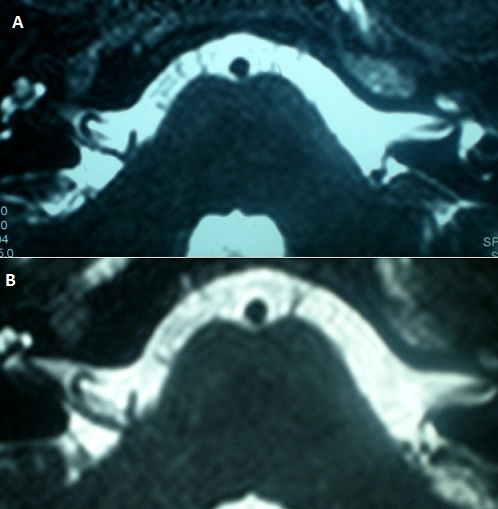
A) IRM des CAI en coupes axiales volumiques T2 –CISS montrant une boucle vasculaire de l'AICA sous croisant à angle droit le trajet du nerf cochléo-vestibulaire au niveau du pore acoustique; B) IRM des CAI en coupes axiales T2 montrant une compression du nerf cochléovestibulaire par une boucle de l'AICA

